# The Antimicrobial Potential of the Neem Tree *Azadirachta indica*

**DOI:** 10.3389/fphar.2022.891535

**Published:** 2022-05-30

**Authors:** Marina R. Wylie, D. Scott Merrell

**Affiliations:** Department of Microbiology and Immunology, Uniformed Services University of the Health Sciences, Bethesda, MD, United States

**Keywords:** neem (*Azadirachta indica* A. Juss), antibacterial, antiviral, antifungal, antiparasitic, phytochemicals, natural products, antibiofilm

## Abstract

*Azadirachta indica* (A. Juss), also known as the neem tree, has been used for millennia as a traditional remedy for a multitude of human ailments. Also recognized around the world as a broad-spectrum pesticide and fertilizer, neem has applications in agriculture and beyond. Currently, the extensive antimicrobial activities of *A. indica* are being explored through research in the fields of dentistry, food safety, bacteriology, mycology, virology, and parasitology. Herein, some of the most recent studies that demonstrate the potential of neem as a previously untapped source of novel therapeutics are summarized as they relate to the aforementioned research topics. Additionally, the capacity of neem extracts and compounds to act against drug-resistant and biofilm-forming organisms, both of which represent large groups of pathogens for which there are limited treatment options, are highlighted. Updated information on the phytochemistry and safety of neem-derived products are discussed as well. Although there is a growing body of exciting evidence that supports the use of *A. indica* as an antimicrobial, additional studies are clearly needed to determine the specific mechanisms of action, clinical efficacy, and *in vivo* safety of neem as a treatment for human pathogens of interest. Moreover, the various ongoing studies and the diverse properties of neem discussed herein may serve as a guide for the discovery of new antimicrobials that may exist in other herbal panaceas across the globe.

## Medicinal Plants as Sources for Novel Antimicrobial Agents

The need to expand the available pharmaceutical repertoire is underlined by several recent reports, including the 2019 Antibiotic Resistance Threat Report by the Centers for Disease Control and Prevention; this document states that in the United States alone, more than 2.8 million antibiotic-resistant infections and more than 35,000 related deaths occur each year (CDC, 2019). These fatal infections are most frequently caused by the 18 species of bacteria and fungi listed as current urgent, serious, or concerning human health threats (CDCE, 2019). Additionally, on a global scale, infectious diseases cause approximately 20% of all deaths each year and are the leading cause of death of children under 5 years old (Martens and Demain, 2017). Many in the medical field agree that devastating statistics like these are a consequence of entering the “post-antibiotic era,” a time in which the efficacies of antibiotics and other antimicrobials are unreliable (Wang et al., 2020; Streicher, 2021).

Despite the continuous popularity of herbal medicine across the globe, traditional antibiotics have previously overshadowed the exploration of plant-based products as therapeutics. However, due to the growing need for new antimicrobial agents, many scientists have now expanded their searches to include novel plant and other environmental sources. Indeed, mainstream medicine is increasingly receptive to the use of plant-derived drugs, especially those to which antimicrobial resistance is more difficult or unlikely to develop. Notably, 26% of all new approved drugs and 33% of all new small-molecule approved drugs between 1981 and 2014 were botanical drugs, unaltered natural products, or derivatives thereof (Newman and Cragg, 2016). This abundance underscores the vast, untapped potential of plants around the world to yield desperately needed novel drugs. In fact, only around 6% of the ∼300,000 species of higher plants have been pharmacologically investigated (Cragg and Newman, 2013). However, recent reviews by Khameneh et al. (2019) and Chassagne et al. (2020) have highlighted the increasingly evident antibacterial properties of various plant species and phytochemicals. Furthermore, there is an increasing amount of evidence that suggests that phytochemicals may be used in conjunction with current antimicrobials to obtain synergistic effects (Aiyegoro and Okoh, 2009; Borges et al., 2016; Barbieri et al., 2017; Ayaz et al., 2019). For example, an early study by Ahmad and Aqil (2007) found that crude extracts of multiple plant species showed *in vitro* synergistic activity with existing antibiotics when used against two multidrug-resistant enteric bacterial species (Ahmad and Aqil, 2007). Thus, the combination of phytochemicals and antibiotics may help to combat resistance to conventional monotherapies for many diseases. As evidence for the use of natural products to treat human disease continues to accumulate, it will become increasingly important to perform in-depth safety studies on the identified extracts, compounds, and their derivatives. So far, there is a general consensus that natural products, as compared to synthetic drugs, have relatively low toxicity to mammals and have less harmful effects on nontarget beneficial organisms (Brahmachari, 2004), which is yet another appealing aspect of utilizing plant species for the identification of effective pharmaceuticals.

## The Importance of *Azadirachta indica* (Neem) as a Medicinal Plant

*Azadirachta indica* (A. Juss), commonly known as the neem tree, is a tropical evergreen tree that is native to the Indian subcontinent (Noorul Aneesa, 2016). For thousands of years, neem has been recognized for its wide array of beneficial properties, including those in agriculture for pest control and in traditional medicine for various common human ailments. *A. indica* originally provoked world-wide interest due to its capacity as a non-toxic infection-control agent for use in farming (Govindachari, 1992). Indeed, one of the most abundant compounds found within the neem plant, azadirachtin, is an increasingly common biopesticide (Chaudhary et al., 2017; Pasquoto-Stigliani et al., 2017; Kilani-Morakchi et al., 2021). However, various parts of the neem tree have been used for millennia in traditional Indian medicine for their claimed antipyretic, antacid, antiparasitic, antibacterial, antiviral, antidiabetic, contraceptive, antidermatitic, anticancer, anti-inflammatory, antioxidant, antifungal, dental, and other healing and protective properties (Govindachari, 1992; Alzohairy, 2016). Almost every part of *A. indica* (e.g., the stem, bark, roots, leaves, gum, seeds, fruits, flowers, etc.) have been used as house-hold remedies for human illnesses. Moreover, millions of people globally use neem twigs as a source of chewing sticks for dental hygiene (Brahmachari, 2004; Gupta et al., 2017). More recently, the neem tree has gained attention from modern medicine and infectious disease researchers as a potential source for new antimicrobials, in addition to the applications of *A. indica* in the fields of oncology, dentistry, dermatology, and endocrinology, among others; for reviews on some of these individual topics, see (Lakshmi et al., 2015; Patel et al., 2016; Aumeeruddy and Mahomoodally, 2021; Iman et al., 2021; Patil et al., 2021; Singh et al., 2021; Yarmohammadi et al., 2021).

Subsequent sections of this review seek to provide an overview of the most recent scientific findings that support the consideration of neem extracts and phytochemicals as antimicrobial agents. Specifically, the potential for neem and neem-related products to target pathogens that are resistant to first-line antibiotics, bacterial species that affect oral health and/or form difficult to eradicate biofilms, fungal infections that threaten food sources, and viral infections that have major impacts on human health are highlighted. Research in these fields is supported by a worldwide interest in neem products that stretches from ancient medicinal practices to an abundance of publications that highlight *A. indica* as a plant with modern pharmacological attributes. Apart from the activities highlighted herein, we would like to point the reader to the expert review by Saleem et al. (2018) that covers the most recent evidence supporting neem as a treatment for specific human ailments and known mechanisms of action of neem components; therein, the specific protective qualities of *A. indica* and related clinical trials are thoroughly reported.

## Phytochemistry of *Azadirachta indica*

Although nearly every part of neem has been used for traditional medicinal purposes in India, the most widely available *A. indica* product on the market today is neem oil (Sir and Chopra, 1994). The country of India alone produces hundreds of thousands of tons of neem oil annually (National Research Council, 1992) and a byproduct of neem oil production includes neem cake, which is abundantly used in agriculture around the world at ∼600 pounds per acre of farmland (Reddy, 2020). Neem oil is considered a vegetable oil that is cold pressed from the fruits and seeds of neem (Noorul Aneesa, 2016). However, neem oil can be further processed into various types of extracts, via different solvents, that are then used for subsequent preclinical and clinical studies.

Although various solvents can be implemented to extract different active components from plant products, most of the compounds that are thought to be responsible for the biological activities of neem can be found in the extracts that are typically used in laboratories (e.g., water, ethanol, methanol, chloroform, and ether) (Cowan, 1999). In recently published literature, methanol and ethanol extracts are those that are most commonly used for antimicrobial testing. The general biological activities of the tested neem oil extracts have been attributed to the presence of many secondary plant metabolites, which include classes of compounds such as isoprenoids (e.g., terpenoids containing limonoid structures) and non-isoprenoids (e.g., tannins) (Saleem et al., 2018).

The neem tree contains hundreds of compounds (i.e., phytochemicals), many of which have been found to be bioactive and to have diverse utility on their own. Out of the more than 300 unique compounds have been identified within the neem tree, some of the more abundant phytochemicals (e.g., azadirachtin, gedunin, and nimbolide) have already been defined as potential drugs with a wide range of biological activities (Saleem et al., 2018; Braga et al., 2020; Nagini et al., 2021). The compounds that have been most thoroughly investigated for their individual antimicrobial properties thus far are limonoids, which are compounds that typically consist of four, six-membered rings and one five-membered aromatic ring (i.e., a furanolactone core) and make up one-third of the phytochemicals derived from the neem tree (Roy and Saraf, 2006; Gupta et al., 2017). This class of compounds has also been explored for its abundance of antioxidant activities [reviewed in (Tundis et al., 2014; Gualdani et al., 2016; Sarkar et al., 2021)] and includes nimbolide, nimbin, and nimbidin (triterpenoids), azadirachtin, and gedunin. Previously established *in vitro* activities of these compounds and others isolated from neem seed oil have been reviewed and range from anti-inflammatory and antiulcer to spermicidal and anti-psoriasis (Brahmachari, 2004). Two recent comprehensive reviews by Saleem et al. (2018) and Gupta et al. (2017) have expertly covered the extensive phytochemistry of neem.

## Antimicrobial Testing and Safety of *Azadirachta indica*

To test the antimicrobial activities of neem oil extracts and phytochemicals, *in vitro* methods such as broth dilution, disc or agar diffusion, and agar overlay assays are commonly used to determine the minimum inhibitory concentration (MIC) and minimum bactericidal concentration (MBC) of each treatment. A few *in vivo* models have been implemented to more accurately reflect human infection and disease testing; these models include intraperitoneal or intravenous injection, or oral or gastric administration of neem oil-related drugs in mice, rats, guinea pigs, and rabbits. These published animal studies indicate that the acute toxicity level of neem greatly depends on the plant component and solvent used to make the extract, as well as on the treatment route and species used in the model; for a comprehensive review on this topic, see (Braga et al., 2021). As an example, oral administration of an ethanolic neem leaf extract less than 2000 mg/kg body weight did not cause mortality in mice (Kanagasanthosh et al., 2015). Conversely, the ethanolic extract of neem stem bark given to rats at 50–200 mg/kg altered the biochemical markers of toxicity and may have consequential effects on organ function (Ashafa et al., 2012). In contrast, one small human study showed that the lyophilized powder of an aqueous neem bark extract given at doses of 30–60 mg twice daily for 10 weeks had therapeutic potential in adults for controlling gastric hypersecretion and gastroesophageal and gastroduodenal ulcers; there were no obvious effects on blood parameters indicative of organ toxicity (Bandyopadhyay et al., 2004). Also, after 1 year of external exposure to 1% neem oil, 156 adults and 110 children did not experience any major adverse effects (Brahmachari, 2004). Of note, Arsene et al. (2021) showed that the *Galleria mellonella* (wax moth larva) model is a reliable method to assess the acute *in vivo* toxicity of medicinal plants, in which ethanolic extracts of neem leaves and seeds had higher levels of toxicity than aqueous extracts of the same materials. Although the available *in vivo* data will need to be further developed before neem oil extracts and phytochemicals are applied in a clinical setting, the United States Environmental Protection Agency has stated that cold-pressed neem oil should have “no unreasonable adverse effects to the US population and the environment” (Agency U.S E.P, 2012). For neem-derived products, non-aqueous extracts are generally the most toxic, while unprocessed materials and pure phytochemicals from the neem tree have relatively low toxicities (Boeke et al., 2004). Given the currently available information summarized here, an important goal of future antimicrobial testing of neem oil and its products should be the standardization of extracts and administration methods. Additional more consistent studies on this topic may allow researchers to draw more detailed conclusions about the potential use of *A. indica* in a clinical setting.

## Antibacterial Evidence

The rising rates of antibiotic resistance for bacterial pathogens has led to the need for novel therapeutics; thus, much of the recent work on the antimicrobial potential of neem has focused on the antibacterial properties of the plant. This area of research is supported by the traditional use of neem products for dental hygiene and the successful applications of neem in the food industry. In addition to standard antibiotic resistance, the ability of pathogenic bacterial species to form biofilms has led to an increased interest in describing how these communities contribute to heightened tolerance to antibacterial substances. Although the importance of biofilm-associated infections to human disease is well-recognized, few novel solutions that effectively eliminate biofilms have been developed thus far. However, encouraging data suggest that neem is consistently more effective at prohibiting bacterial growth and at targeting biofilm-grown cells than many other herbal extracts and is, therefore, worth pursuing as a source for drug discovery (Noor, 2011). For a more comprehensive list of the antibacterial properties of the neem tree described in the following sections, see Supplementary Table S1.

### *Azadirachta indica* and Dentistry

As mentioned above, the use of neem twigs as dental cleaning sticks is commonplace in many countries to which *A. indica* is a native species (Gupta et al., 2017). This traditional use has translated to a growing number of studies that have tested neem products for their ability to improve dental hygiene and to prevent or treat oral diseases. Typically, these studies aim to identify an application for neem extracts as a mouthwash alternative, root canal irrigant, toothpaste, etc. Previously, the antimicrobial activity of *A. indica* against common endodontic pathogens, such as *Enterococcus faecalis*, *Staphylococcus aureus*, *Streptococcus mutans*, and *Candida albicans*, has been well-established (Mistry et al., 2014; Barua et al., 2017; Singh et al., 2020). For example, a decade ago, it was shown that at 7.5%, aqueous neem leaf extract was able to inhibit the growth of *E. faecalis*, *S. mutans*, and *C. albicans* and that the MIC of an ethanolic neem leaf extract was 1.88%, 7.5%, and 3.75% against these three important dental pathogens, respectively (Nayak Aarati et al., 2011). More recently, another group was able to determine that a methanolic extract of *A. indica* showed considerable antimicrobial activity against a three-week-old polymicrobial dental biofilm grown on extracted human teeth consisting of *S. mutans, E. faecalis, S. aureus* and *C. albicans* (Mistry et al., 2015). The results from two small human studies indicate that a neem-based toothpaste or gel can reduce the levels of *S. mutans* in the mouth and that the gel formulation can reduce plaque and gingivitis to the same degree as a chlorhexidine gel control (Nimbulkar et al., 2020; Selvaraj et al., 2020). Additional biologically relevant studies may be able to provide even more evidence for the use of neem in dentistry in combination with, or as an alternative to, antimicrobials already used in the field. Of note, several studies have already suggested that neem extracts have similar levels of activity as chlorhexidine or hypochlorite (typical components of oral washes) against plaque, gingivitis, and pain *in vivo* (Jalaluddin et al., 2017; Hosny et al., 2021) and against biofilm-forming bacteria (e.g., *Streptococcus viridans, Porphyromonas gingivalis,* and *S. aureus*) and *C. albicans in vitro* or *ex vivo* (Joy Sinha et al., 2015; Anand et al., 2016; Kankariya et al., 2016; Heyman et al., 2017; Andonissamy et al., 2019; Bansal et al., 2019; Tasanarong et al., 2021). Another human pathogen that causes plaque and other biofilm-related diseases in the body, *E. faecalis*, is also just as susceptible to various neem extracts as it is to chlorhexidine *in vitro* (Chandrappa et al., 2015; Mustafa, 2016; Bhardwaj et al., 2017; Joy Sinha et al., 2017).

The relevance of neem as an antimicrobial in dentistry is, so far, the most researched area and has led to many conclusions about *A. indica* extracts as compared to those from other plants used in traditional medicine. In fact, some studies suggest that neem has greater antibacterial activity than *Commiphora myrrha* (myrrh), *Acacia* tree (e.g., catechu), *Cinnamomum verum* (cinnamon), *Salvadora persica* (miswak), *Syzygium aromaticum* (clove), *Zingiber officinale* (ginger), *Allium sativum* (garlic) and *Curcuma longa* (tumeric) extracts against some species of bacteria and cultured dental caries (Kanth et al., 2016; Jagannathan et al., 2020; Arora et al., 2021). This being said, it is important to note that certain bacterial species (e.g., *S. mutans* and *E. faecalis*) appear to be more susceptible to extracts from other plants in some studies (Jain et al., 2015; Dedhia et al., 2018; Kalita et al., 2019; Panchal et al., 2020). While this does not diminish the antimicrobial potential of *A. indica*, it does underline the importance of thoroughly taking advantage of the wide variety of antimicrobial plants and compounds that are at the disposal of modern medicine.

### The Use of *Azadirachta indica* in the Food Industry

Originally introduced around the world as a potent pesticide and fertilizer for use in agriculture (Govindachari, 1992; Nicoletti et al., 2012; Chaudhary et al., 2017), neem has been recognized in more recent years as a safe and effective broad-spectrum antimicrobial with uses throughout the food industry that range from food production and storage to packaging and human consumption. During meat production, the presence of several species of bacteria can affect the quality and safety of the product, including *Campylobacter*, *Lactobacillus*, and *Carnobacterium* spp. Neem cake extract, which is a waste product from neem seed oil production, has antibacterial activities against all of these potentially pathogenic species (Del Serrone et al., 2015a). Additionally, Ravva and Korn (2015) found that neem leaf and bark supplements were able to successfully eliminate *Escherichia coli* O157:H7 from cultured cow manure; because this *E. coli* strain was isolated from an apple juice outbreak of O157:H7, these results may have broad applications on farms where crops and orchards frequently exist in close proximity to cattle (Ravva and Korn, 2015). In the production of another source of protein for human consumption, specifically in shrimp aquaculture, antibiotic-resistant *Vibrio parahaemolyticus* can compromise both shrimp and human health. A thorough study on the potential use of neem in this industry included *in vitro* and *in vivo* assays that showed that aqueous neem extract had a MIC against *V. parahaemolyticus* of 62.5 mg/ml and was able to significantly increase survival of shrimp by 76% as compared to the untreated control (Morales-Covarrubias et al., 2016).

One of the next steps in the production of pathogen-free human food is storage and/or packaging. In the last couple of years, several groups have shown that food preservation films that are made from polyethylene or sustainable materials such as seaweed can be manufactured to incorporate neem leaf extracts, neem oil, and other plant-based products (e.g., turmeric and curcumin) (Ahmed et al., 2022). The resulting composite films are shelf-stable, block ultraviolet light, and have increased antifungal and antibacterial activities against *C. albicans* and a wide range of Gram-negative and Gram-positive organisms, including *E. coli*, *S. aureus*, *Pseudomonas aeruginosa*, and *Bacillus subtilis* (Sunthar et al., 2020; Uthaya Kumar et al., 2020; Oyekanmi et al., 2021; Subbuvel and Kavan, 2021). Furthermore, the ability of *A. indica* to prevent activity of food-spoiling fungi is evident by several recent reports that outline the following: 1) the ability of neem oil to prevent the growth of the grape product-spoiling species *Aspergillus carbonarius* and to inhibit the production of mycotoxin by strains of this fungus (Rodrigues et al., 2019), 2) the ability of neem leaves to prevent the production of aflatoxins by *Aspergillus parasiticus* during long-term storage of rice, wheat, and maize (Sultana et al., 2015), 3) the ability of neem seed methanol and ethanol extracts to inhibit *Aspergillus flavus* and *A. parasiticus* by 10% in the context of maize storage (An et al., 2019), 4) the ability of multiple neem seed, bark, and leaf extracts to inhibit the growth of three major potato-spoiling fungi, *Aspergillus niger*, *Fusarium oxyporium*, and *Pythium* spp., by 72–100% (Ezeonu et al., 2019), and 5) the ability of aqueous neem leaf extract to inhibit growth of *A. niger* and *A. parasiticus*, as well as to detoxify aflatoxin B1 and ochratoxin A *in vivo* (Hamad et al., 2021). These diverse antifungal properties of neem are highlighted in Supplementary Table S1. Altogether, these food-related studies should encourage further investigation of the utility of *A. indica*-derived products throughout the food industry; these products may serve as a sustainable antimicrobial alternative that can potentially improve food security via improved stable long-term storage, as well as improve human health through the elimination of foodborne pathogens.

### Additional Antibacterial Activities of Neem

In several other areas of antibacterial discovery, *A. indica* has been shown to be effective against many important human pathogens. Overall, a large number of studies have been published in the last decade on this topic, especially as they relate to the ever-growing number of antibiotic-resistant organisms. In this vein, several bacterial species that cause wound infections are found on the long list of antibiotic resistant threats, including *S. aureus* and *P. aeruginosa* (CDC, 2019). Several groups have tested the antibacterial activity of neem against these species. For example, Garg et al. (2015) demonstrated that methanol and chloroform neem extracts performed better than other plant extracts and better than several different antibiotics against both *S. aureus* and *P. aeruginosa* (Garg et al., 2015). More specifically, it has also been determined that the MIC of limonoid compounds that were isolated from neem seeds are between 32 μg/ml and 128 μg/ml against *P. aeruginosa* and another opportunistic skin pathogen, *Staphylococcus epidermidis* (Lu et al., 2019). As a translational approach to this area of research, several studies have demonstrated that an aqueous neem leaf extract that was used to make alginate fibers for wound dressings (Hussain et al., 2017), a nanofibrous mat embedded with neem leaf extract (Ali et al., 2019), and a topical gel containing a methanolic neem extract all inhibit *S. aureus* growth (Raju and Jose, 2019). In the case of a polyesteramide synthesized from neem oil to produce a nanofibrous mat, the incorporation of *A. indica* into this wound treatment method resulted in increased tissue regeneration in rats, as compared to the control commercial cream (Killi et al., 2019). These results potentially have broad applications for many areas of medicine in which the risk of antibiotic resistant skin/wound infections is high.

**FIGURE 1:**
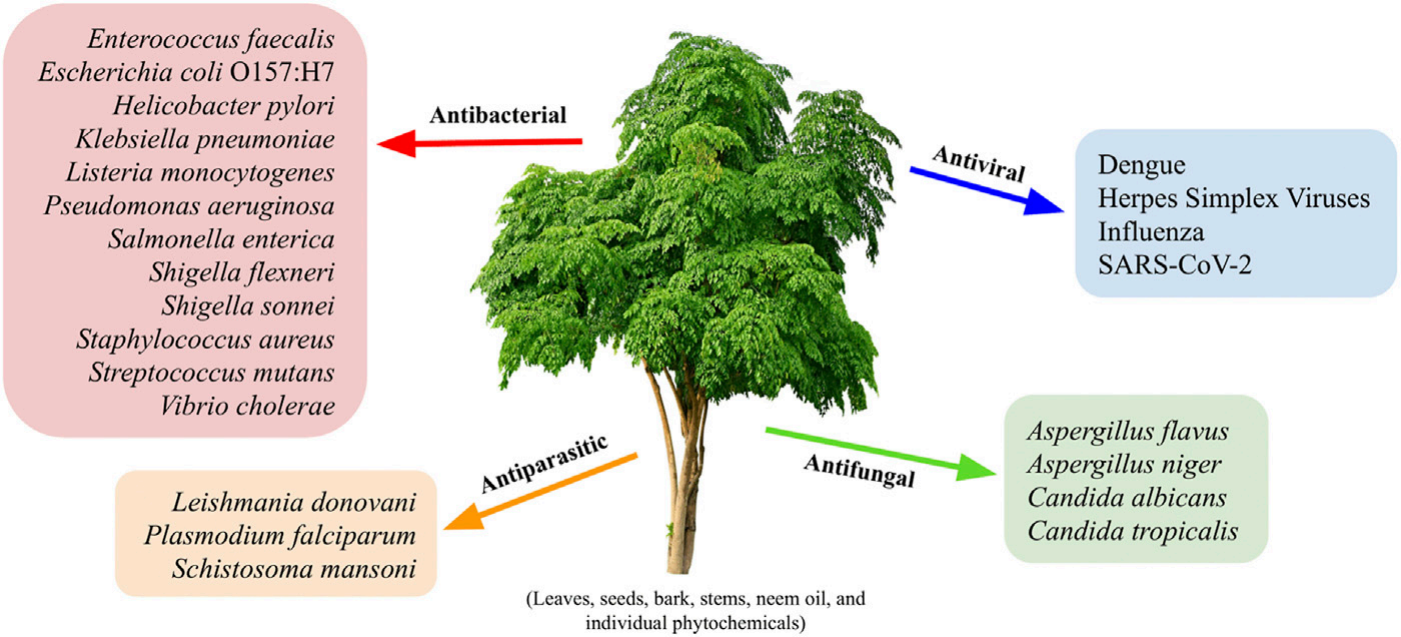
Representative antimicrobial targets of the neem tree, *Azadirachta indic*a. Virtually all parts of the neem tree (leaves, seeds, bark, and stems), neem oil, and individual neem-associated phytochemicals have been shown to possess antibacterial, antiviral, antiparasitic, and/or antifungal activities. Some of the pathogens that have been studied and shown to be susceptible to *A. indica*-associated compounds are listed in their respective categories; the length of the provided list indicates the relative amount of published information on each of the four topics. A more exhaustive list of the pathogens that are susceptible to neem-derived products is available in Supplementary Table S1.

Another large group of infectious bacteria that cause morbidity and mortality all over the world are the gastrointestinal pathogens, which include foodborne and diarrhea-causing organisms. Relatedly, some of the traditional uses of neem are antidiarrheal, antacid, and antiulcer; this has led to a large body of research that has investigated the antibacterial properties of neem products against pathogens such as *Salmonella* spp., *Shigella* spp., *E. coli*, *Listeria monocytogenes*, and *Bacillus cereus*, as shown in Figure 1. To summarize, the *Salmonella* spp. and *Shigella* spp. that have been tested, which includes more than a dozen multidrug-resistant isolates from patients suffering from typhoid fever complications, are susceptible to seed, bark, and leaf extracts of neem from either ethanol, methanol, or acetone extraction; in some cases, the activity of neem extract was also found to be greater than that of gentamycin, erythromycin, and other plants used in traditional medicine (Mahfuzul Hoque et al., 2007; Susmitha et al., 2013; Tesso et al., 2015; Melese et al., 2016; Al Akeel et al., 2017; Panchal et al., 2020; Essuman et al., 2021). Similarly, dried leaf, seed, and bark neem extracts in any of the three previously mentioned solvents have significant antibacterial activity against *E. coli*, with the methanolic extract of neem seeds demonstrating the greatest level of activity (Susmitha et al., 2013; Sharma and Nupur, 2014; Melese et al., 2016). Additionally, neem oil was as effective as ciprofloxacin against 48 tested isolates of *E. coli*, 14 of which were diarrheagenic strains (Del Serrone et al., 2015b). Given that there are more than a quarter of a million cases of *E. coli* each year in the United States alone, associated world-wide morbidity is a huge issue for this pathogen (CDCE, 2019). Indeed, resistant *E. coli* infections were found to be responsible for nearly a quarter of all disability-adjusted life years caused by resistant bacterial infections in the European Union in 2015 (Cassini et al., 2019). On the chronic disease spectrum of gastrointestinal illnesses, *Helicobacter pylori* is a pathogen that colonizes approximately 50% of the human population and causes ulcers in millions of people each year; it also causes stomach cancer in ∼1% of people who are infected (Kuipers, 1999; Moodley et al., 2012; Bray et al., 2018). Recently, two studies found that neem oil extract and a neem-associated phytochemical, nimbolide, have potent *in vitro* bactericidal activity against *H. pylori* in liquid cultures and in biofilms (Blum et al., 2019; Wylie et al., 2021). Similarly, another group determined that an ethanolic neem leaf extract has activity against this species as well (Saxena et al., 2021). Overall, studies like those described above provide strong evidence that novel plant-derived treatments, like neem oil extracts and/or the phytochemicals contained therein, may be able to reduce the burden of pervasive organisms like *E. coli* and *H. pylori*.

Finally, there have been many studies published on the antibacterial properties of neem against a variety of diverse human pathogens. The overwhelming conclusion from the majority of these investigations is that many elements of *A. indica* (e.g., seeds, bark, leaves, etc.) produce extracts that have moderate to significant levels of antimicrobial activity against several pathogens: *S. aureus*, *E. coli*, *E. faecalis*, *P. aeruginosa*, *Salmonella typhi*, *Streptococcus agalactiae*, *Shigella boydii*, *B. subtilis*, *Klebsiella pneumoniae*, and *Candida tropicalis*, as listed in Figure 1 (Dahiya and Purkayastha, 2012; Melese et al., 2016; Al Saiqali et al., 2018; Ibrahim and Kebede, 2020). Moreover, in side-by-side comparisons *A. indica*-based components often show superiority to other plants; a few exceptions include aqueous garlic extract and ethanolic green tea extract that were each shown to work better than neem extracts to kill *Bacillus anthracis* or *S. aureus* and *E. coli*, respectively (Zihadi et al., 2019; Kaur et al., 2021). Overall, studies support the notion that *A. indica* is an omnipotent plant that possesses antimicrobial activity against many bacterial pathogens. Future studies should include more standardized research approaches to test the potential of neem-derived products and individual compounds against other microbes both *in vitro* and *in vivo*; a priority should also be placed on determining the mechanisms of action of these products in order to fully understand the ideal clinical significance of *A. indica*.

### Neem and Biofilm-Forming Pathogens

Biofilms, or communities of bacteria composed of biofilm-associated cells and extracellular polymeric substance (EPS) components (e.g., proteins, polysaccharides, and extracellular DNA), are notably recalcitrant to outside stressors, including those from the immune system and from therapeutics (Bae and Jeon, 2013; Yonezawa et al., 2019; de Vor et al., 2020). Depending on the species of biofilm-associated bacteria, mechanisms of tolerance include slow diffusion/limited penetration of the drug, metabolic heterogeneity and decreased growth rates among the cells within the biofilm, and the formation of persister cells (Mah and O'Toole, 2001; Stewart and Costerton, 2001; Attaran et al., 2017). Backed by thorough research, plants such as *A. indica* may provide the tools needed to treat pervasive biofilm infections. To this end, there is evidence that natural products may be an ideal source of quorum sensing inhibitors, efflux pump inhibitors, and metal chelators, which are all potentially powerful antibiofilm agents (Borges et al., 2016). Additionally, synergistic interactions between the many constituents found within plant extracts may provide a benefit over a single isolated ingredient; this may explain the efficacy of lower doses of herbal products like neem oil as compared to individual compounds (Aiyegoro and Okoh, 2009).

Significant biofilm-associated human infections are caused by species such as *S. aureus*, *E. faecalis*, and *P. aeruginosa* (Vestby et al., 2020). There is already evidence that *A. indica* has activity against biofilm-forming strains of some of these pathogens. For example, a neem leaf ethanolic extract was found to inhibit *S. aureus* and methicillin-resistant *S. aureus* (MRSA) biofilm adherence at 62.5 and 125 μg/ml, respectively (Quelemes et al., 2015). More recently, another group found that a petroleum ether neem extract had a MIC and MBC of 125 and 500 μg/ml, respectively, against a strain of MRSA (S et al., 2020). In the same *in vitro* study, the addition of 1 mg/ml of the neem extract resulted in a 68.9% reduction in MRSA biofilm; 2 mg/ml of the same extract resulted in a 83.8% reduction (S et al., 2020). Additionally, Guchhait *et al.* found that ripe neem seed extracts had antibiofilm activity against *S. aureus* and *Vibrio cholerae*. The minimum biofilm inhibitory concentrations (MBIC) and minimum biofilm eradication concentrations (MBEC) for this extract were 100 and 300 μg/ml, respectively, against *S. aureus* and 300 and 500 μg/ml, respectively, against *V. cholerae* (Guchhait et al., 2022). Furthermore, using a mouse model of *V. cholerae* infection, Thakurta *et al.* showed that administration of methanolic neem leaf extract at 100–1800 mg/kg body weight reduced intestinal fluid secretion by 27.7%–77.9% and doses ≥300 mg/kg inhibited *Vibrio*-induced hemorrhage in the murine intestine without signs of toxicity (Thakurta et al., 2007). Of note, individual neem-associated phytochemicals, such as catechin, may have greater potential for biofilm eradication, persister cell damage, disruption of EPS structural components, and prevention of quorum sensing (Lahiri et al., 2021). In addition, a methanolic neem oil extract and nimbolide both killed cells within *in vitro* biofilms produced by the Gram-negative carcinogenic pathogen, *H. pylori* (Wylie et al., 2021). Overall, evidence indicates that neem has great potential to be used as a therapeutic for resistant bacterial infections. However, future research that utilizes animal models will be crucial to determine whether neem-derived products fit in with established antibiotic regimens and/or work alone to eradicate biofilms *in vivo*.

## Antiviral Evidence

Although most recent studies have investigated the antibacterial and antifungal potential of *A. indica*, some work has also explored the antiviral activities of neem; this topic has previously been reviewed in (Dhawan, 2012). To date, most publications have centered around human immunodeficiency virus (HIV), as well as the herpes, Dengue, and influenza viruses; however, recent reports have also included SARS-CoV-2, which is responsible for the COVID-19 pandemic. Moreover, a few groups have successfully experimented with the use of neem products against other viruses, such as Japanese encephalitis virus (Dwivedi et al., 2021b), hepatitis C (Ashfaq et al., 2016), and coxsackie virus (Younus et al., 2016). The antiviral studies have primarily focused on the ability of individual neem-associated phytochemicals to block critical processes of the viral life cycle, including cell entry and replication. Intriguingly, this means that the obtained results may describe a mechanism of action as well as identify a distinct drug candidate that can be directly modified for pharmaceutical development. Finally and importantly, as an alternative method to control specific classes of viral or parasitic diseases, many researchers have demonstrated the ability of *A. indica* derivatives to deter and/or to negatively affect the many species of insect vectors that transmit these pathogens [reviewed in (Benelli et al., 2017a)] (Soni and Prakash, 2014; Abiy et al., 2015; Benelli et al., 2015; Maheswaran and Ignacimuthu, 2015; Poopathi et al., 2015; Chandramohan et al., 2016; Murugan et al., 2016; Yerbanga et al., 2016; Benelli et al., 2017b; Kaura et al., 2019; Paramo et al., 2020; Rasool et al., 2020; Ejeta et al., 2021; Zatelli et al., 2022).

### Neem and HIV

Human immunodeficiency virus, or HIV, is arguably one of the most devastating modern human pathogens. Since its discovery in the early 1980s, there have been over one million new HIV infections each year; hundreds of thousands of people still die from the subsequent acquired immunodeficiency syndrome (AIDS) annually (Barre-Sinoussi et al., 1983; Gallo et al., 1984; Levy et al., 1984; UNAIDS, 2022). Although antiretroviral therapy (ART) is well-established and is successful at diminishing viral load and preventing disease progression, ART drugs are expensive, and require life-long treatment that is not without side effects (CDC, 2022). To this end, natural products, such as those obtained from *A. indica*, that are traditionally used for HIV-associated infections (Nagata et al., 2011; Anywar et al., 2020), have been explored for their abilities to protect the CD4^+^ T cell population that is vulnerable during HIV infection, to reduce persistent immune activation during ART, and to decrease the toxicity of ART drugs. For instance, small trials have concluded that neem leaf extract given daily is safe and effective at improving CD4^+^ T cell counts in HIV patients (Udeinya et al., 2004; Mbah et al., 2007). Furthermore, when *A. indica* and *Senna siamea* leaf extracts were given in combination with ART, this HIV patient group had improved T cell numbers and fewer markers of hepatic and renal toxicity than the group that was given ART alone (Goni Hamad et al., 2021). To address the possibility of T cell exhaustion in HIV-infected patients, Olwenyi et al. (2021) performed an *in vitro* study with peripheral blood cells isolated from infected and uninfected individuals; following exposure to enterotoxin, the lymphocytic response indicated that *A. indica* extract, but not the extracts from two other plants, was able to down-regulate CD4^+^ T cell activation in a concentration-dependent manner without affecting general T cell-specific functions. Overall, these results support the idea that neem has immunomodulatory abilities that can be exploited to increase the efficacies of certain treatments and to improve the condition of chronically infected patients, such as those with HIV.

### Herpes and Sulfonoquinovosyldiacylglyceride From *Azadirachta indica*

Herpes simplex viruses (HSV) most commonly cause oral and genital infections, with an estimated half a billion people living with HSV type 2 and nearly four billion people with HSV type 1 in any given year (James et al., 2020). Because there is no cure for HSV infections and they can recur many times throughout a person’s life, broad searches for novel antiviral medications are warranted in order to identify agents that may reduce the morbidity associated with these infections; these searches include phytochemicals isolated from medicinal plant such as *A. indica*. For example, the glycolipid sulfonoquinovosyldiacylglyceride (SQDG) that has been isolated from neem leaves was shown to have potent antiviral activity against HSV-1 and -2; the half maximal effective concentrations (EC_50_) were 9.1 and 8.5 μg/ml, respectively. The same study also found that HSV-infected, SQDG-treated macrophages produced significantly less proinflammatory cytokines than untreated controls (Bharitkar et al., 2014). It has been suggested that the antiviral and anti-inflammatory properties of SQDG may indicate that *A. indica* contains other phytochemicals with therapeutic potential against viruses (Shanmugam et al., 2020). Concurrently, it was found that two polysaccharides isolated from neem leaves, along with their sulfated derivates, are able to inhibit HSV-1 nucleic acid synthesis at concentrations that were not cytotoxic (Faccin-Galhardi et al., 2019). Additionally, an aqueous neem bark extract was able to block HSV-1 glycoprotein binding to and virus entry into target cells *in vitro* (Tiwari et al., 2010). The preliminary data reported here indicate that products derived from *A. indica* can act at several steps of the viral life cycle to prevent herpes infections.

### *Azadirachta indica* Components Against Dengue Proteins

Several groups have demonstrated that computational screening methods can be successfully used to identify novel inhibitors of viruses that infect hundreds of millions of people globally each year, including vector-borne diseases such as Dengue (CDC, 2021). In the context of Dengue virus (DENV), 49 different bioflavonoids that are present in the neem tree were virtually screened for binding to the DENV serine protease, NS2B-NS3pro. Subsequent *in vitro* assays with promising candidates revealed that kaempferol 3-O-β-rutinoside and epicatechin were able to inhibit DENV-2 infectivity by 77.7% and 66.2%, respectively, without significant cell toxicity (Dwivedi et al., 2021a). Similarly, it was previously shown that three members of another important class of neem phytochemicals, the terpenoids, were able to bind to NS2B-NS3pro with high affinity *in silico*; this binding ability was subsequently confirmed *in vitro* (Dwivedi et al., 2016). Nimbin, one of the more common triterpenoids isolated from neem leaf extracts, was shown to be effective against the envelope protein of all four types of DENV *in silico* (Lavanya et al., 2015). Overall, the ability of neem-derived phytochemicals to block the activities of both the protease and envelope proteins of DENV, and potentially other viruses, further suggest that *A. indica* may be a novel source for antiviral drugs (Shanmugam et al., 2020).

### Influenza and Neem Phytochemicals

Flu leads to an estimated 290,000-650,000 deaths annually (WHO, 2020). Due to these consistently high levels of associated morbidity and mortality around the world, influenza is one of the most intensely researched viruses. Though new vaccines for influenza are constantly in development, the burden of flu could be additionally reduced by the introduction of an antiviral that is effective at all stages of disease. Recent evidence suggests that *A. indica* may represent a robust source of novel drugs against viruses such as influenza. To summarize, molecular docking experiments identified a total of four neem phytochemicals that interact with conserved residues of either the nucleoprotein or the non-structural (NS1) protein of influenza. Though requiring further testing, this may indicate the ability to act as a universal drug against the flu virus (Ahmad et al., 2015; Ahmad et al., 2016).

### *Azadirachta indica* and SARS-CoV-2

Taking the lives of more than five million people over the last 2 years, SARS-CoV-2 and the associated disease, COVID-19, continue to be significant threats to public health for which there are few treatment options (WHO, 2021b). Consequently, many researchers have successfully screened chemical libraries for viral inhibitors that act against SARS-CoV-2 (Garg et al., 2020; Vardhan and Sahoo, 2020; Baildya et al., 2021; Borkotoky and Banerjee, 2021; Gogoi et al., 2021; Nallusamy et al., 2021; Navabshan et al., 2021; Senapati et al., 2021; Ogidigo et al., 2022; Vardhan and Sahoo, 2022); detailed reviews on the potential for medicinal plants to be used against SARS-CoV-2 are available (Thota et al., 2020; Adithya et al., 2021). Phytochemicals and plant-derived products like those from *A. indica* are sometimes included in these chemical libraries. For example, *in silico* binding simulations by Parida et al. (2020) identified nimolicinol as a compound with strong affinity for both the main protease and the spike protein of SARS-CoV-2; similar docking studies found that nimbocinol binds to the papain-like protease of SARS-CoV-2 with higher affinity than remdesivir, indicating its potential to hinder viral replication (Balkrishna et al., 2021). Although these types of studies are high throughput and can detect candidates for further investigation, virtual studies must typically be followed by *in vitro* experiments before an antiviral can have clinical relevance. Despite this, given the devastation associated with the ongoing pandemic, several clinical trials are in progress to test neem as a component of mouthwashes, nasal sprays, and capsules for suspected, confirmed, or hospitalized COVID-19 patients, or for prophylaxis (Khan et al., 2020; Farhan Raza Khan, 2021; Nesari et al., 2021). Additionally, one animal study has been completed in which coronavirus-infected mice were treated orally or intranasally with neem bark extract; overall, the therapy was able to prevent systemic injury and pathologic effects of the virus both *in vivo* and *in vitro* using multiple model systems (Sarkar et al., 2022). Advocating for additional trials of neem against SARS-CoV-2, Eze et al. (2022) made connections between some of the known mechanisms of *A. indica* products against other illnesses and the potential efficacy of neem against the virus that causes COVID-19. For additional information about the pharmacological implications for the use of medicinal plants against respiratory infections, see the recent review by Timalsina et al. (2021). All in all, the antiviral action of *A. indica* against a variety of viruses that cause human disease is evident (Supplementary Table S1). Moreover, outside of HIV, HSV, DENV, influenza, and SARS-CoV-2, there is a strong indication that the aforementioned phytochemical screening methods may be used to identify novel inhibitors of other viral life cycles.

## Antiparasitic Evidence

Although the area of research regarding *A. indica* as a potential therapeutic against parasites is relatively underdeveloped, most of the investigations that have been published so far have focused on *Plasmodium*, the human pathogen of global importance that causes malaria; more than half a million deaths attributed to malaria are estimated to occur each year (WHO, 2021c). While there are effective prophylactics and therapeutics approved for this disease, these resources are not readily available in all parts of the world. In these instances, there is the possibility that natural products may be more easily acquired, distributed, and accepted; neem products could be part of the solution. Recently, a comprehensive review on the antiplasmodial activities of African medicinal plants was published and found that neem extracts consistently performed well across experiments (Tajbakhsh et al., 2021). Studies using both *in vitro* and *in vivo* models indicate that aqueous, methanolic, or ethanolic extracts of neem stems, leaves, or bark all have significant antimalarial activity against a variety of *Plasmodium falciparum* and *Plasmodium berghei* strains (Benoit et al., 1996; MacKinnon et al., 1997; El-Tahir et al., 1999; Gathirwa et al., 2011; BC et al., 2013; Tepongning et al., 2018). These publications suggest that *A. indica* and other medicinal plants are active against malaria; however, only one clinical trial with an African plant species has been conducted so far (Benoit-Vical et al., 2003). Despite the lack of controlled human studies, the malaria mouse model has been used to demonstrate that neem bark and seed extracts are active against *Plasmodium* infection both alone and as part of a polyherbal mixture; in the case of the bark extract, this was indicated by parasite suppression or decreased erythrocyte infection (Habluetzel et al., 2019; Alaribe et al., 2021). The gestational malaria mouse model has also been used to show that administration of *A. indica* leaves improves the overall health of *P. falciparum*-infected dams, including reduced parasitemia, increased platelet counts, lower levels of preeclampsia biomarkers, and increased birth weight of pups (Amadi et al., 2021). Additionally, the limonoid deacetylnimbin, which is found within neem seed extracts, is able to interfere with the early sporogony stages of *P. bergehi*; this finding suggests that certain neem-associated phytochemicals may have the ability to limit *Plasmodium* transmissibility, warranting further investigation in other models and clinical trials (Tapanelli et al., 2016). Another commonly studied phytochemical of *A. indica*, azadirachtin, was shown to bind to Gephyrin E almost as well as artesunate during *in silico* analyses, which indicates that, upon further study, azadirachtin may be an effective treatment for cerebral malaria (Okoh et al., 2021). Providing additional convincing data for the investigation of neem products as therapeutics, Somsak et al. (2015) used the mouse model of *Plasmodium* infection to show that an aqueous neem leaf extract was able to reduce the blood markers of malaria-induced renal injury to normal levels without being toxic to the animals. Taken together, the available *in vitro* and *in vivo* data suggest that plants used in traditional medicine (e.g., neem) should be further explored in clinical trials for their antimalarial capacity.

Throughout tropical and subtropical areas around the world, the neglected tropical protozoan leishmania causes an estimated 700,000 to 1 million infections each year and is highly fatal if left untreated; visceral leishmaniasis is the most severe form of associated disease (WHO, 2021a). Leishmaniasis can be treated, but there is not currently a drug available that eliminates the leishmania parasite from the body; thus, the patient is susceptible to relapse if immunosuppression occurs (WHO, 2021a). Although studies on this topic are limited, there is some evidence that plant extracts can kill leishmania parasites, including an ethyl acetate fraction (EAF) of neem leaf extract (Dayakar et al., 2015). When treated with this EAF, promastigotes underwent an apoptosis-like death and intracellular amastigotes were also killed *in vitro* and *in vivo* (Dayakar et al., 2015). With this evidence, more *in vivo* and translational research on the antileishmanial activity of neem is merited.

Finally, the last antimicrobial activity of *A. indica* that has been recently explored is that against parasitic worms (Figure 1). As the most widespread neglected tropical diseases globally, the burden of schistosomiasis and soil-transmitted helminth infections could undoubtedly be reduced with the addition of new and accessible plant-derived treatments or prophylactics to the already available drug regimens (Molyneux et al., 2017). Although still a minor area of research, the antischistosomal and anthelminthic properties of neem suggest that natural products can be potent inhibitors of larger eukaryotic pathogens as well. Indeed, the same neem leaf extract that was effective against *S. aureus* and MRSA biofilms also caused severe tegument morphology changes, significant reduction of motor activity, or death of *Schistosoma mansoni* worms *in vitro* (Quelemes et al., 2015). For helminth related studies, an *in vivo* experiment showed that neem leaf powder used at 500 mg/kg body weight worked as well as 5 mg/kg fenbendazole to treat cows with bovine *Strongyloides* infections (Jamra et al., 2015); *Strongyloides* is a major hurdle to profitable farming in tropical and subtropical regions and can have a substantial economic impact (Jamra et al., 2015). Similarly, problematic helminths for the poultry industry are *Raillietina* spp*.*, which are parasitic tapeworms. These organisms were found to be severely paralyzed, damaged, or killed by short exposures to SQDG, a glycolipid from neem extracts that also has antiviral activity (Ash et al., 2017). Although not yet tested on human helminth infections, neem-derived products appear to have strong potential against animal pathogens, thus supporting the need for a deeper investigation of the overall antiparasitic activity of *A. indica*.

## The Future of Neem as an Antimicrobial

As summarized in this review, a variety of extracts and phytochemicals from the Indian evergreen tree, *A. indica*, have significant antimicrobial activity against a multitude of pathogens that affect human health. The dental application of neem appears to be one of the most researched areas, with the general antibacterial properties of neem not far behind. Intriguingly, there are several recent studies that suggest that neem may have broad applications in the food industry, aside from its traditional uses in agriculture for pest-control and fertilizer. Some of the least-developed areas, however, are the antiviral and antiparasitic activities of neem-derived products. Overall, there is sufficient evidence to warrant further investigation into these properties of *A. indica*; undoubtedly, *in vivo* models will be crucial to understanding the clinical relevance of neem against all of the microorganisms mentioned here and those that have yet to be tested.

Of note in recent years, there are several groups who have incorporated neem into novel materials and technologies that have broad implications for human health. Specifically, green-synthesized copper or silver nanoparticles and hydrogels, nanocellulose films, chitosan-copper oxide biopolymers, and hydroxyapatite have all been constructed to include neem extracts and have substantial antimicrobial activity, including against multidrug-resistant bacterial species (Nagaraj and Samiappan, 2019; Revathi and Thambidurai, 2019; Algebaly et al., 2020; Asghar and Asghar, 2020; Lakkim et al., 2020; Sharma and Bhardwaj, 2020; Ulaeto et al., 2020; Chinnasamy et al., 2021; Ghazali et al., 2022; Lan Chi et al., 2022). Both *in vitro* and *in vivo* data suggest that these composite materials represent a growing industry of creative antimicrobial technologies that have the potential to revolutionize infectious disease treatments and biomedical science as a whole.

In addition to the many recently published manuscripts that indicate that *A. indica*-derived substances have broad-spectrum antimicrobial properties, it is worth noting that dozens of patents are filed each year that mention neem-based products. Indeed, a simple query of the United States Patent and Trademark Office’s online Patent Public Search tool (USPTO, 2022) using the terms “neem and antimicrobial” yields over 400 results since 2015. This available list of patents helps to demonstrate the limitless applications of the antimicrobial properties of neem. While too many exist to cover in detail here, some notable examples include very diverse applications. For example, medical gloves (Wong et al., 2022) and a polymeric yarn for use in hygienic textiles (Mandawewala and Mandawewala, 2021) have both been enriched with neem derivates and shown to have beneficial antimicrobial properties. Furthermore, in line with the reported antimicrobial uses of neem in the fields of dentistry, dermatology, and agriculture, many of the patented neem-containing products fall into these categories as well; patents have been awarded for neem-containing dental rinses and composites (Tatch, 2021; Ramana, 2022), for neem and/or other plant extract-containing topical treatments for mild skin disorders (Balaraman, 2015), and for neem-based pest control formulas (Mazariegos, 2016). Clearly, the investigation of neem as an antimicrobial is an area of research that is constantly expanding and is generating valuable products that may improve human health.

In order to develop realistic *A. indica*-based treatment regimens that could be used in humans, there are clearly many intriguing areas for future investigation. Undoubtably, future experiments will need to elucidate the mechanisms of action of neem and the associated phytochemicals. Given the available data summarized in this review, some of the most promising areas of investigation moving forward appear to be 1) the application of individual neem phytochemicals and derivatives thereof as antimicrobial agents alone or used in combination with existing treatments, 2) additional pre-clinical and clinical studies to determine the toxicity and effective *in vivo* dosing of specific phytochemicals as compared to parent neem extracts, and 3) the inclusion of hundreds of available medicinal plant products, extracts, and phytochemicals in screens for potential inhibitors of emerging and resistant infectious diseases. It is important to note that to gain maximal utility from these areas of research, close attention should be paid to the types of extracts (including both the particular part of the plant and the solvent) that have already been tested against which organisms. Some level of standardization should be considered so that comparisons can be made and patterns can be recognized across multiple studies. This may become easier when the antimicrobial activities of more individual phytochemicals are determined. *En masse*, *A. indica* represents a novel source of antimicrobials that may be used to combat drug resistance and emerging threats to human health. Furthermore, the research that has been done on the neem tree can be used as a guide to encourage the investigation of other traditionally used natural products for their utility as modern pharmaceuticals.

## Abbreviations

AIDS, acquired immunodeficiency syndrome; DENV, Dengue virus; EAF, ethyl acetate fraction; EC50, half maximal effective concentration; EPS, extracellular polymeric substance; HIV, human immunodeficiency virus; HSV, herpes simplex virus; MBC, minimum bactericidal concentration; MBEC, minimum biofilm eradication concentration; MBIC, minimum biofilm inhibitory concentration; MIC, minimum inhibitory concentration; MRSA, methicillin-resistant *Staphylococcus aureus*; SQDG, sulfonoquinovosyldiacylglyceride.
